# The Salt Tolerance Related Protein (STRP) Mediates Cold Stress Responses and Abscisic Acid Signalling in *Arabidopsis thaliana*

**DOI:** 10.3389/fpls.2020.01251

**Published:** 2020-08-13

**Authors:** Anna Fiorillo, Maurizio Mattei, Patrizia Aducci, Sabina Visconti, Lorenzo Camoni

**Affiliations:** Department of Biology, University of Rome Tor Vergata, Rome, Italy

**Keywords:** salt tolerance related protein, cold stress, abscisic acid, *Arabidopsis thaliana*, late embryogenesis abundant proteins

## Abstract

Low temperature stress is one of the major causes of crop yield reduction in agriculture. The alteration of gene expression pattern and the accumulation of stress-related proteins are the main strategies activated by plants under this unfavourable condition. Here we characterize the *Arabidopsis thaliana* Salt Tolerance Related Protein (STRP). The protein rapidly accumulates under cold treatment, and this effect is not dependent on transcriptional activation of the *STRP* gene, but on the inhibition of proteasome-mediated degradation. Subcellular localization of STRP was determined by the transient expression of *STRP-YFP* in *A. thaliana* protoplasts. STRP is localized into the cytosol, nucleus, and associated to the plasma membrane. Under cold stress, the membrane-associated fraction decreases, while in the cytosol and in the nucleus STRP levels strongly increase. STRP has high similarity with WCI16, a wheat Late Embryogenesis Abundant (LEA)-like protein. Despite no canonical LEA motifs in the STRP sequence are present, physicochemical characterization demonstrated that STRP shares common features with LEA proteins, being a high hydrophilic unstructured protein, highly soluble after boiling and with cryoprotectant activity. To clarify the physiological function of STRP, we characterized the phenotype and the response to low temperature stress of the *strp* knockout mutant. The mutation causes an equal impairment of plant growth and development both in physiological and cold stress conditions. The *strp* mutant is more susceptible to oxidative damage respect to the *wild type*, showing increased lipid peroxidation and altered membrane integrity. Furthermore, the analysis of Abscisic acid (ABA) effects on protein levels demonstrated that the hormone induces the increase of STRP levels, an effect in part ascribable to its ability to activate *STRP* expression. ABA treatments showed that the *strp* mutant displays an ABA hyposensitive phenotype in terms of seed germination, root development, stomata closure and in the expression of ABA-responsive genes. In conclusion, our results demonstrate that STRP acts as a multifunctional protein in the response mechanisms to low temperature, suggesting a crucial role for this protein in stress perception and in the translation of extracellular stimuli in an intracellular response.

## Introduction

Low temperature is a major environmental factor that adversely affects survival, growth and development of plants, thus limiting their geographical distribution and affecting productivity and quality of crops (Sanghera et al., 2011). Over the course of evolution, plants have evolved a repertoire of physiological and biochemical responses to cope with cold stress, including alteration of metabolism, changes in membrane lipid composition, production of osmolytes and release of reactive oxygen species (ROS). These responses protect plants and are crucial for the acclimation to the low temperatures. Cold acclimation is a dynamic process that occurs at different levels, including the activation of specific subset of cold-responsive (*COR*) genes, mainly mediated by C-repeat (CRT)-binding factors (CBFs) transcription factors, also known as dehydration-responsive element (DRE)-binding proteins (DREBs), (Liu et al., 1998; Jia et al., 2016; Zhao et al., 2016). These transcription factors are rapidly induced by low temperature and recognize a specific CRT/DRE cis-element in the promoter of *COR* genes (Shi et al., 2018), that include genes encoding for enzymes of lipid metabolism and osmo-protectants biosynthesis, late embryogenesis abundant (LEA) proteins, transcription factors and proteins involved in hormone signalling (Liu et al., 2019). However, many *COR* genes lack the CRT/DRE sequence, supporting the existence of a CBF-independent regulation for these genes, during cold acclimation (Park et al., 2015; Zhao et al., 2016). The CBF-independent pathway is also mediated by both ABA-dependent and ABA-independent mechanisms (Huang et al., 2017). ABA is a key phytohormone in the regulation of many physiological processes and plays a crucial role in stress response (Verslues and Zhu, 2005; Fujii et al., 2009; Kim et al., 2016). Although ABA levels only slightly increase under cold stress (Lang and Palva, 1992), different evidence supports the involvement of this hormone in cold acclimation. About 10% of ABA-responsive genes are also activated by low temperature (Kreps et al., 2002), plants acquire freezing tolerance when treated with exogenous ABA (Kumar et al., 2008; Kim et al., 2016), and the ABA-deficient mutant *aba-1* has cold acclimation impaired ability (Koornneef et al., 1982; Heino et al., 1990). In addition, several proteins induced by low temperature are also synthesized in response to ABA (Cattivelli and Bartels, 1992; Palva, 1994), suggesting that hormone biosynthesis and signalling are important for *COR* genes activation (Gilmour and Thomashow, 1991; Mantyla et al., 1995). In the last years, evidence for COR-independent pathways have been also provided (Liu et al., 2019). Nevertheless, these pathways remain largely uncharacterized.

Cold stress responses involve changes in the cellular protein profile and, in this background, several proteomic studies identified a multitude of differently represented proteins in different plant species subjected to low-temperature regimes (Bae et al., 2003; Amme et al., 2006; Li et al., 2011; Rocco et al., 2013).

Rocco et al. (2013) identified in *Arabidopsis thaliana* leaves 28 proteins whose levels were affected by short-term cold stress. Among these proteins, our attention was focused on a 16 kDa protein (TAIR accession At1g13930) whose levels are greatly increased (13-fold) under cold stress. The function of this protein is unknown, but it was related with salt tolerance. In fact, the amino acid sequence is 59% identical to ST6-66, a *Thellungiella halofila* protein that confers salt tolerance when overexpressed in *A. thaliana* (Du et al., 2008). Accordingly, the *loss of function* mutant is hypersensitive to salt stress, hence the protein name Salt Tolerance Related Protein (STRP) was proposed (Du et al., 2008).

Different pieces of evidence propose the involvement of STRP in the response mechanisms to abiotic stresses. The protein has a 42% of amino acid sequence similarity with WCI16, a wheat Late Embryogenesis Abundant (LEA)-like protein that plays a role in freezing tolerance (Sasaki et al., 2014). Moreover, STRP was found to interact with DEK3, a nuclear protein involved in chromatin remodelling processes in response to salt stress (Waidmann et al., 2014).

The goal of the present study was to unveil the physiological function of STRP and to clarify its role in the plant response to cold stress. To this purpose, STRP subcellular localization and the mechanism underlying protein accumulation in response to cold stress have been investigated. Moreover, the effect of *STRP* mutation was analysed both at the biochemical and physiological level, pointing out a crucial role of STRP in cold stress response.

## Materials and Methods

### Plant Materials

The *A. thaliana* plants used in this study were of Columbia (Col-0) ecotype. The *strp* knockout mutant (SALK_076125; Alonso et al., 2003) was obtained from the Nottingham Arabidopsis Stock Center (NASC).

### Growth Conditions and Phenotype Analysis

*Wild type* and *strp* mutant seedlings were grown in soil in a growth chamber at 22° C, 80% humidity, under a 16/8 h light/dark cycle. Plants were subjected to cold stress by incubation at 4° C for 30 min-18 h periods, maintaining the same light conditions. Hypocotyl length was measured by harvesting at least 30 seedlings after 7 days. Photographs were taken using a digital camera and the acquired images were processed using ImageJ (Schneider et al., 2012). Leaf area was measured by analysing digital pictures with Easy Leaf Area software (Easlon and Bloom, 2014).

For MG132 (Selleckchem, Houston, TX) and ABA treatments, *wild type* or *strp* seeds were sterilized with 70% ethanol and a 1% sodium hypochlorite solution containing 0.05% Tween-20, washed with sterile water and sowed on Murashige and Skoog (MS) medium containing 1% sucrose and 0.8% agar, pH 5.7. Seeds were stratified at 4° C in the dark for 3 days, and then transferred in the growth chamber at 22° C, 80% humidity, under a 16/8 h light/dark cycle for 2 weeks. Plants were then transferred on MS supplemented with 100 µM MG132 or different ABA concentrations. Stressed and control plants were then sampled and subjected to further analysis, as described below.

### Germination Assays

To assess the effects of ABA on *strp* seeds germination, 200 *wild type* and *strp* seeds were sowed on MS medium supplemented with different ABA concentration. After stratification, seeds were transferred to a growth chamber and the germination rate assessed after one week. The germination rate in the absence of stratification was determined on 200 *wild type* and *strp* seeds sowed on MS and immediately transferred to a growth chamber.

### ABA-Mediated Regulation of Plant Growth

For ABA-mediated inhibition of primary root elongation, lateral roots formation and plant growth rate, 200 *wild type* and *strp* seeds, sowed and germinated on MS, were transferred after 4 days to the same medium supplemented with different ABA concentrations. After one-week, primary root was measured by analysing digital pictures with ImageJ software, while the number of lateral roots was determined by counting the roots with a stereomicroscope. The inhibition of plant growth was assessed after two weeks by measuring the seedling fresh weight with an analytical balance.

### Water-Loss and Stomatal Aperture Assays

For water-loss assay, 25 two-week-old *wild type* and *strp* seedlings were transferred in MS plates containing 20 µM ABA. After 18 h, leaves were detached, and their weight was recorded 6 times every 15 min. Stomatal aperture measurements were done in leaves of three-week-old *wild type* and mutant lines as described by Yu et al. (2016). Leaves were incubated for 2 h with the stomata open buffer (SOB, 10 mM Mes, 50 mM KCl, 0.1 mM CaCl_2_, pH 6.15) and transferred to the SOB containing 20 µM ABA. After 2 h incubation, the leaf lower epidermis was detached by sticking to adhesive tape (Hansen and van Ooijen, 2016), stained for 20 min with 30 µM propidium iodide and observed under a fluorescence microscope (Nikon TE 2000, Nikon Instruments Inc., Melville, NY). Propidium iodide fluorescence was detected exciting with an Argon laser at 488 nm and emission was detected in the 600–680 nm range. At least 50 stomata were measured in each experiment.

### Gene Expression Analysis

Total RNA was extracted from 100 mg of *A*. *thaliana* leaves using the RNeasy Plant Mini Kit RNA from Qiagen (Germantown, MD). Two micrograms of RNA were used for retro-transcription with FastGene Scriptase II cDNA-Kit (Nippon Genetics Europe, Düren, Germany). qPCR was performed in triplicates by using specific qPCR primers (Sigma-Aldrich Corporation, St. Louis, MO), the qPCRBIO SyGreen Mix (PCR Biosystem, London, UK) and the Real-Time PCR Light-Cycler II (Roche Diagnostics, Indianapolis, IN). mRNA levels were normalized to actin (*ACT8*), GAPDH and tubulin beta chain (*TUB8*) transcripts, and the relative mRNA levels were determined by using the 2-ΔΔCt method (Pfaffl, 2001). The primer sequences are listed in **Supplemental Table S1**.

### Protoplast Purification and Transformation

The STRP coding sequence (Accession Number AT1G13930) was cloned upstream of the YFP in a modified pGreen 0029 binary vector (Zottini et al., 2008). STRP cDNAs was inserted in the *NcoI* restriction site located at the 5’ end of YFP sequence using appropriate primer pairs (**Supplemental Table S1**). *A. thaliana* protoplasts were isolated and transformed as described by Yoo et al. (2007). For transient transformation, 20 µg of plasmid and 10^4^ protoplasts were added to an equivalent volume of a solution containing 40% PEG 4000, 0.2 M mannitol, 100 mM CaCl_2_. Samples were incubated for 30 min at 23° C, diluted with W5 buffer (2 mM MES-KOH, 154 mM NaCl, 125 mM CaCl_2_, 5 mM KCl, pH 5.7), centrifuged at 800 g for 1 min and resuspended in W5 buffer. After 18 h at 23° C incubation, transformed protoplasts were imaged on GFP fluorescence by confocal microscopy with a laser scanning microscope (Olympus FV1000). Lasers 488 nm (Argon) and 635 nm (diode) were used to detect the green and red fluorescence of YFP and chlorophyll signals, respectively. Cell Mask Orange and Hoechst 33342 (Thermo Fisher Scientific, Waltham, MA), detected with lasers at 554 nm and 361 nm, were used for plasma membrane and nuclei staining, respectively. Images were acquired using a 40× objective.

### STRP Recombinant Protein and Antiserum Production

To produce the recombinant protein, the coding sequence of STRP was amplified by PCR using appropriate primer pairs (**Supplemental Table S1**) and cloned into the pGEX-2T vector. Recombinant protein was expressed in *E. coli* as a GST-fusion protein, affinity purified using Glutathione Sepharose 4B (GE Healthcare, Chicago, IL) and the GST was removed by thrombin digestion.

For polyclonal anti-STRP antibodies production, 1 mg purified STRP was mixed with Freund’s complete adjuvant (1:1, v/v) and injected into two rabbits. Two booster injections were given at 2-week intervals. Antisera were then used for Western blot analysis.

### Cell Free Degradation Assay

The cell free degradation assay was performed accordingly to Osterlund et al. (2000). Two-week-old *A. thaliana* plants were homogenised to a fine powder with liquid nitrogen and resuspended in 20 ml of degradation buffer (25 mM Tris-HCl, 10 mM NaCl, 10 mM MgCl_2_, 10 mM ATP, 1 mM PMSF, 5 mM DTT, pH 7.5). Cellular debris were removed by two-centrifugation steps at 17000 g at 4° C for 10 min. The supernatant was recovered, and samples were incubated with MG132 50 µM (Selleckchem, Houston, TX) for 15, 30, and 60 min at 22° C. Samples were then subjected to SDS-PAGE and Western blot analysis.

### Preparation of Plant Whole Cellular Extract

*A. thaliana wild type* and *strp* seedlings were homogenised in liquid nitrogen and the powder was resuspended in 2.5 ml of extraction buffer (25 mM Tris-HCl, 1 mM EDTA, 150 mM NaCl, 10% glycerol, 5 mM DTT, 2 mM PMSF, 2% polyvinylpyrrolidone, 0,1% Nonidet P-40, pH 7.5). Samples were centrifuged at 8,000 g for 30 min at 4° C to completely remove the cell debris. The supernatant was collected, and protein concentration determined with the Bradford method (Bradford, 1976), using BSA as a standard.

### Isolation of Plasma Membrane, Cytosol and Nuclei

Cytosolic fraction and two-phase partitioned plasma membranes were isolated from *A.* *thaliana* leaves as previously described (Pallucca et al., 2014). Nuclei were isolated as described by Xu and Copeland (2012). Briefly, 8 g of leaves from three-week-old *A. thaliana* plants were grinded in a cold mortar. The powder was resuspended in 20 ml of Lysis Buffer (20 mM Tris-HCl, 25% glycerol, 20 mM KCl, 2 mM EDTA, 5 mM MgCl_2_, 250 mM sucrose, 1 mM DTT, and 1 mM PMSF, pH 7.4). The homogenate was sequentially filtered through a 100 μm and a 40 μm nylon mesh and centrifuged at 1,500 g for 10 min at 4° C. The pelleted nuclei were first washed in 3 ml Nuclei Resuspension Buffer (NRB, 20 mM Tris-HCl, 25% glycerol, 2.5 mM MgCl_2_, pH 7.4) containing 0.2% Triton X-100, then in 3 ml NRB buffer and finally resuspended in 400 µl NRB containing 440 mM sucrose.

Soluble nuclear protein and chromatin binding fractions were isolated from 400 µl nuclear suspension as described by Barbosa et al. (2010). Pelleted nuclei were treated with 100 μl of Nuclear Lysis Buffer (NLB, 10 mM Tris-HCl, 0.5% sodium deoxycholate, 10 mM NaCl, 3 mM MgCl_2_, 10 mM NaF, 1% Tween-20, 1.2 mM PMSF). Soluble nuclear proteins were separated from chromatin by centrifugation at 20,000 g for 5 min at 4° C. After 2 washes in NLB, the chromatin fraction was incubated in 50 μl Digestion Buffer (10 mM Pipes, 50 mM NaCl, 300 mM sucrose, 3 mM MgCl_2_, 1 mM EGTA, 10 mM NaF, 0.5% Triton X-100, 1.2 mM PMSF, 0.6 U/μl DNase I, 50 ng/µL RNase A) for 1 h at 20° C and then treated for 5 min at room temperature with 5 µl ammonium sulphate 2.5 M. Chromatin-associated proteins were separated by centrifugation at 20,000 g for 10 min at 4° C.

### Electrolyte Leakage and Lipid Peroxidation Assays

The electrolyte leakage assay was performed as described by Visconti et al. (2019). *A. thaliana* leaves (0.2 g) were cut into 5 mm slices and incubated in 30 ml of deionized water under continuous stirring for 2 h at 25° C. The electrical conductivity of solution was measured by using an electrical conductivity meter. Boiled samples were used to determine the maximum percentage of electrolyte leakage. Lipid peroxidation was evaluated by measuring malondialdehyde (MDA) production, following the procedure described by Taulavuori et al. (2001).

### Protein Solubility Assay and Cryoprotection of LDH Activity

To determine the STRP protein solubility, samples were boiled for 20 min, centrifuged at 12,000 g for 20 min and separated by SDS–PAGE. The cryoprotective activity of recombinant STRP was assayed as described by Sasaki et al. (2014) using L-Lactic Dehydrogenase from rabbit muscle (Sigma-Aldrich Corporation).

### Western Blot

For immunoblotting analysis, samples were separated by SDS-PAGE, performed as described by Laemmli (1970) in a Mini Protean apparatus (Bio-Rad, Hercules, CA), and transferred onto a PVDF membrane with a Bio-Rad semi-dry apparatus following the manufacturer’s instructions. Anti-STRP polyclonal antibody 1:10,000 dilution, anti-H3 (Sigma-Aldrich Corporation) 1:2,000 dilution, anti-H^+^-ATPase (Camoni et al., 2006) 1:2,000 dilution and anti-actin (Sigma-Aldrich Corporation) 1:1,000 were used. Immunoblot detection was performed by incubating the membrane with horseradish peroxidase conjugated anti-rabbit secondary antibody (1:5,000) or anti-mouse secondary antibody (1:5,000) from Bio-Rad.

### Hydropathy Plot Analysis and DynaMine Prediction

Hydropathy analysis of STRP, based on Kyte–Doolittle values (Kyte and Doolittle, 1982), was performed by using ExPASy tool (Gasteiger et al., 2005). Protein dynamics was predicted by using the DynaMine webserver (Cilia et al., 2013).

### Statistical Analysis

Each experiment was performed at least three times. Statistical significance was assessed by unpaired Student’s t-test. All values are expressed as means ± S.E.M.

## Results

### Effect of Cold Stress on STRP Levels and Sub-Cellular Localization

**Figure 1:**
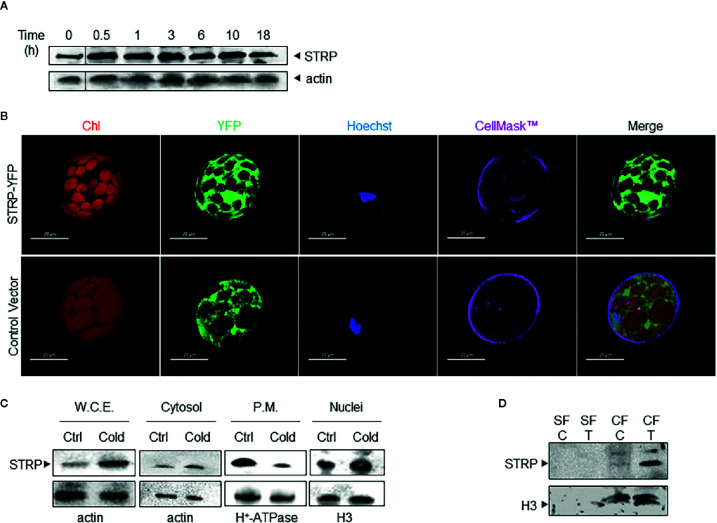
Cold stress affects STRP subcellular localization and levels. **(A)**: time course of STRP levels under cold stress. *A. thaliana* whole cellular extract was prepared after 0.5, 1, 3, 6, 10, and 18 h of cold stress. 15 μg of samples were separated by SDS-PAGE and subjected to western blotting with anti-STRP antibodies. Actin was used as loading control. **(B)**: STRP subcellular localization in *A. thaliana* mesophyll protoplasts expressing STRP-YFP fusion protein. CellMask™ Orange and Hoechst 33342 were used as marker for plasma membrane and nuclei, respectively. YFP signal is shown in green, chlorophyll in red, Hoechst 33342 in blue and CellMask™ Orange is pseudo-coloured in magenta. Scale bar, 20 μm. **(C)**: 15 µg of proteins from whole cellular extract (W.C.E.), cytosol, plasma membrane (P.M.) and nuclei were separated by 12% SDS-PAGE and analysed by western blotting with anti-STRP antibodies. Actin, H^+^-ATPase and H3 were used as loading control. **(D)**: analysis of STRP levels in the soluble nuclear fraction (SF) and in the chromatin fraction (CF) obtained from control (C) and cold treated (T) nuclei. 20 µg of SF and CF were separated by SDS-PAGE, blotted onto PVDF membrane and immunodecorated with anti-STRP antibodies. Histone H3 was used as marker for CF.

The identification of STRP as a cold up-regulated protein (Rocco et al., 2013) points to its possible role in cold stress response mechanisms. To deeper assess the effect of cold stress on STRP levels, three-week-old *A. thaliana* plants were exposed to 4° C for periods ranging from 30 min to 18 h. Whole cellular extracts were prepared and analysed by western blot using polyclonal anti-STRP antibodies, obtained by immunizing a rabbit with STRP recombinant protein (**Figure S1**). The time course shows that STRP is rapidly accumulated under cold stress conditions, being protein levels already increased after 30 min of stress (**Figure 1A**).

In order to study the subcellular localization of STRP, mesophyll protoplasts were purified from *A. thaliana* leaves and transiently transformed with the 35S-STRP-YFP construct. STRP is localized into the cytosol, in the nucleus and it is also associated to the plasma membrane (**Figure 1B**). To assess whether cold stress brings about variations on STRP subcellular localization, cytosolic, nuclear and plasma membrane fractions were purified from cold-treated *A. thaliana* plants, and protein levels analysed by western blot. As shown in **Figure 1C**, cold stress induces a strong decrease of STRP levels associated to the plasma membrane, whilst protein content in the cytosolic and nuclear fraction is greatly increased.

The recent identification of STRP as an interactor of the DEK Domain-Containing Protein 3 (DEK3), a nuclear protein involved in chromatin remodelling, suggested that in the nucleus STRP can be associated to chromatin. To test this hypothesis, soluble and chromatin-bound proteins were purified from nuclei and analysed by western blot. As shown in **Figure 1D**, STRP is entirely associated with the chromatin fraction. Interestingly, the amount of STRP bound to chromatin-greatly increases under cold stress.

### Effect of Cold Stress on STRP Stability

**Figure 2:**
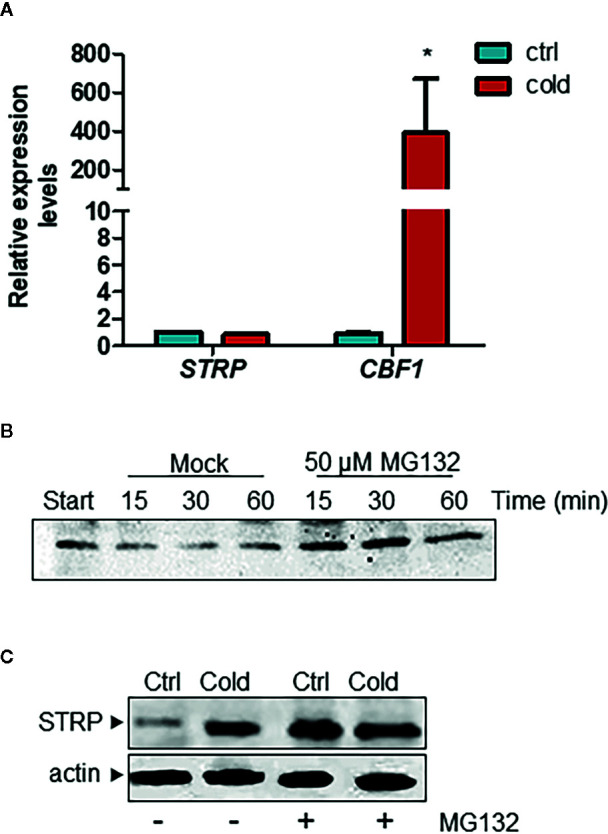
Cold stress stabilizes STRP protein by inhibiting its proteasome-mediated degradation. **(A)** *STRP* expression levels under cold stress was determined on total RNA extracted from *A. thaliana* plants exposed 6 h at 4°C. RNA was retrotranscribed and analysed by RT-qPCR. mRNA levels were normalized to GAPDH mRNA. Error bars are S.E.M. of three independent experiments. *P < 0.01, by Student’s t-test. **(B)** cell-free degradation assay: 15 µg of total extract were treated with 50 µM MG132 or DMSO for 15, 30 and 60 min and then analysed by western blotting with anti-STRP antibodies. **(C)** effect of MG132 on STRP levels. 20 µg of cytosol extracted from control (Ctrl) and cold-exposed (Cold) plants treated with MG132 for 18 h were separated by SDS-PAGE, transferred on PVDF membrane and immunodecorated with anti-STRP antibodies. Actin was used as loading control.

In order to ascertain whether the increase of STRP levels under cold stress is due to a transcriptional activation of the *STRP* gene, a RT-qPCR was performed on plants exposed to cold stress for 6 h. As shown in **Figure 2A**, *STRP* is not activated by cold stress, whereas the cold responsive gene *CBF1*, used as positive control, results to be greatly activated. A similar mechanism that leads to STRP accumulation in the absence of gene expression activation has been also observed in response to salt stress (Du et al., 2008; Fiorillo and Camoni, personal communication). This finding suggests that STRP levels could be regulated by post-transcriptional mechanisms leading to protein stabilization under different abiotic stress. In order to ascertain whether STRP can be target of a proteasome-dependent degradation mechanism in response to cold stress, a cell-free degradation assay was performed. Proteins were extracted from two-week-old *A. thaliana* plants, incubated at 22° C for 15, 30 and 60 min with the 26S proteasome inhibitor MG132 and then analysed by western blotting. As shown in **Figure 2B**, STRP is rapidly degraded in mock-treated samples, whereas MG132 brings about STRP stabilization. To investigate whether cold stress affects STRP levels by regulating protein stability, *A. thaliana* seedlings, grown on MS medium, were transferred on MS supplemented with 100 µM MG132 and exposed at 4° C for 18 h. As shown in **Figure 2C**, STRP levels strongly increase in the presence of MG132 in unstressed samples, equalling the levels of the cold-treated one. This result suggests that, in control conditions, STRP is an unstable protein targeted for the 26S proteasome-dependent proteolysis, and that its degradation is inhibited by cold stress.

### Physicochemical Characterization of STRP

**Figure 3:**
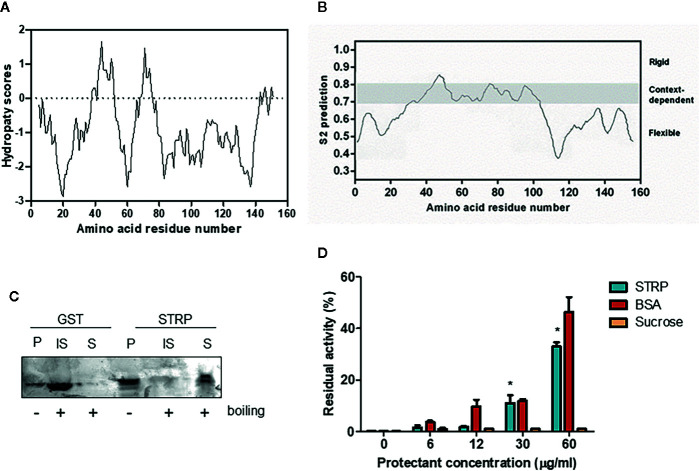
STRP physicochemical properties. **(A)** hydropathy plot of STRP. The analysis was performed by using Kyte–Doolittle values (Kyte and Doolittle, 1982) with a window size of 9. Negative values indicate the predicted hydrophilic regions. **(B)** prediction of STRP folding with DynMine system (Cilia et al., 2013). **(C)** STRP solubility after boiling. Recombinant STRP and GST were treated (+) or not (−) for 20 min at 100° C, cooled and separated by SDS-PAGE. P, purified protein; IS, insoluble fraction; S, soluble fraction. **(D)** STRP cryoprotection assay on LDH. LDH was frozen for 24 h at −20° C in presence of different concentrations of cryoprotectants. The enzyme was thawed on ice and the residual activity, expressed as a percentage of enzyme initial activity before the freeze-thaw cycle, measured at 340 nm. Error bars are S.E.M. of three independent experiments. *P < 0.01, by Student’s t-test.

STRP has no similarity to other *A. thaliana* proteins (Du et al., 2008). The hydropathy plot, obtained using the Kyte–Doolittle algorithm (Kyte and Doolittle, 1982) shows that STRP is a highly hydrophilic protein, with few short hydrophobic regions (**Figure 3A**). Given the amino acid sequence similarity between STRP and the wheat LEA-like protein WCI16 (Sasaki et al., 2014), the possibility that STRP could share some biochemical properties with WCI16 was investigated. The protein backbone dynamics was analysed with the DynaMine prediction system. This system analyses the amino acid sequence of the proteins, in order to identify folded, unfolded and disordered regions. As shown in **Figure 3B**, STRP is highly unstructured and flexible, with only a small portion able to assume a stable conformation in context-dependent manner. Accordingly, the analysis of conserved protein domains, carried out with the NCBI Conserved Domain Search -NIH (Marchler-Bauer et al., 2017), revealed that the protein has no conserved known domains (data not shown). The lack of a defined secondary structure explains why the STRP apparent molecular mass on SDS–polyacrylamide gels is 25 KDa, greater than the expected mass of 16 KDa (**Figure S1**). In fact, intrinsically unstructured proteins bind less SDS than other proteins do, and consequently their electrophoretic mobility is reduced (Tompa, 2002).

Several LEA proteins are comprised in a more widespread group, called hydrophilins (Garay-Arroyo et al., 2000; Battaglia et al., 2008). The physicochemical characteristics that define this group are a Gly content greater than 6% and a hydrophilicity index (Kyte and Doolittle, 1982) greater than 1. STRP has a Gly content of 9% and a hydrophilicity index of 0.88, a value that does not allow to identify STRP as a hydrophilin. However, similar values are in any case typical of some LEA proteins families.

Based on these results, other typical features of LEA proteins, such as the solubility after boiling and the cryoprotectant activity, were investigated. STRP solubility after boiling was assessed on recombinant STRP using GST as a control. Proteins were boiled for 20 min and subsequently cooled on ice. The soluble fraction was separated from the insoluble one, and proteins were analysed by SDS-PAGE. As shown in **Figure 3C**, STRP is fully soluble after boiling, whereas GST is found in the insoluble fraction. This feature is typical of unstructured and highly hydrophilic proteins, where no hydrophobic regions are exposed to the solvent after boiling. On the contrary, structured proteins, like GST, expose the hydrophobic regions, causing aggregation and precipitation.

The cryoprotectant activity was assessed by measuring the ability of STRP to prevent the inactivation of the freeze-labile enzyme lactate dehydrogenase by freezing treatment. Equivalent concentrations of Bovine Serum Albumin (BSA) and sucrose were used as positive and negative control, respectively. As shown in **Figure 3D**, 30 and 60 µg/ml STRP preserve LDH activity similarly to BSA. This result highlights a cryoprotectant activity for STRP, thus suggesting the possibility that STRP may function preventing damage of cellular enzymes in stress conditions.

### *strp* Mutant Characterization

In order to clarify the physiological function of STRP and to ascertain its role in cold stress responses, the characterization of the *STRP* knock-out mutant was carried out. The *strp* mutant utilized (SALK_076125) is characterized by a T-DNA insertion into the STRP promoter (Baulcombe et al., 1986). The genotype of the STRP mutant was determined by means of a genomic three-oligonucleotides PCR assay (Du et al., 2008). The mutant is homozygous for the T-DNA insertion (**Figure S2A**). The absence of *STRP* expression in the mutant was confirmed by RT-PCR and RT-qPCR (**Figure S2B**). Western blot analysis shows that STRP protein is undetectable in whole cellular extract from two-week-old plants, thus confirming that the STRP insertional mutant is not able to synthetize the protein (**Figure S2C**).

**Figure 4:**
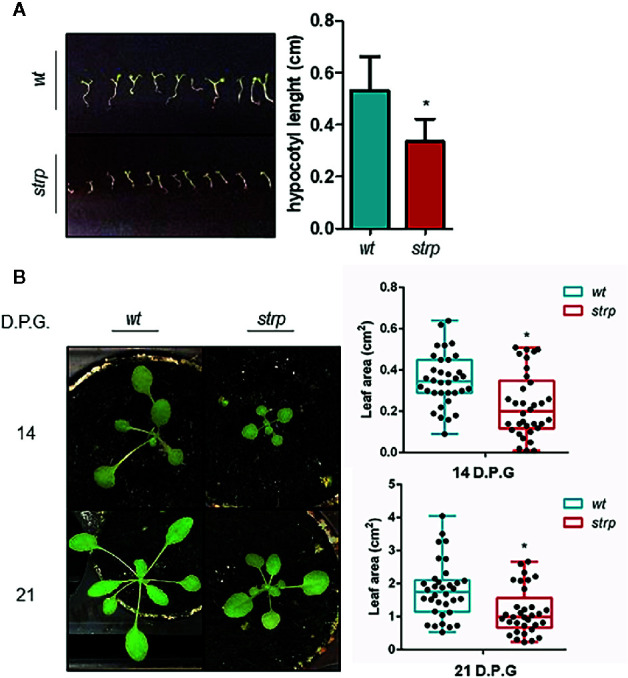
Phenotypical characterization of *strp* mutant. **(A)** hypocotyl length of one-week-old seedlings. Hypocotyls were measured by using ImageJ (Schneider et al., 2012). **(B)** rosette leaf area of plants grown for one, two or three weeks in universal soil. Rosette area was determined with EasyLeaf area software (Easlon and Bloom, 2014). D.P.G., days post-germination. Error bars are S.E.M. of four independent experiments. *P < 0.01, by Student’s t-test.

To evaluate whether the *strp* mutation causes an impairment of plant growth and development, the phenotypical analysis in physiological conditions was performed. One-week post-germination, hypocotyls length was measured. As shown in **Figure 4A**, the *strp* mutant has shorter hypocotyls compared to *wild type* plants. Rosette leaf area was measured after two- and three-weeks post- germination. As shown in **Figure 4B**, the rosette of the *strp* mutant is smaller compared to the *wild type*. Hence, *STRP* *loss of function* mutation produces a significant impairment of plant growth in physiological conditions.

In order to ascertain whether cold stress conditions can induce more severe growth defects on the *strp* mutant compared to *wild type* plants, three-week-old plants were exposed for 5 nights to a 4° C for 8 h, in parallel with the photoperiodic cycle.

**Figure 5:**
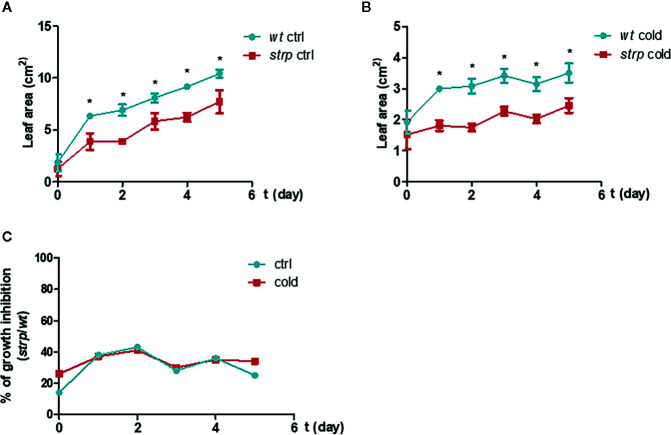
Cold stress affects *wild type* and *strp* plant growth. Rosette leaf area was determined on plants grown in universal soil for three weeks and then exposed to low temperature, during the night, for additional 5 days. Rosette area was determined with EasyLeaf area software. Panel **A**: ctrl; Panel **B**: cold treated; Panel **C**: percent ratio of growth inhibition between *strp* and *wild type* plants. Error bars are S.E.M. of four independent experiments. *P < 0.01, by Student’s t-test.

As shown in **Figure 5**, the *strp* mutant has a smaller rosette as compared to *wild type* plants, both in control (**Figure 5A**) and cold stress conditions (**Figure 5B**). However, the reduction rate of leaf area of the *strp* mutant is similar both in control and cold stress conditions (**Figure 5C**). Therefore, overall data highlight that *strp* mutation brings about a significant growth impairment, which is not however more severe under cold stress.

### Biochemical Responses to Cold Stress in the *strp* Mutant

Since a strong increase of STRP levels was observed under cold stress conditions, a protective role for this protein was hypothesized. Under cold stress, high amounts of ROS are released, generating a severe oxidative burst (Dreyer and Dietz, 2018). The most common and well-characterized damages caused by ROS occur at the level of biological membranes. Therefore, the content of malondialdehyde (MDA), an oxidation product of the unsaturated membrane lipids, indicator of membrane structural integrity (Hodges et al., 1999), was evaluated. MDA production was measured on two-week-old plants exposed to 4° C for 18 h.

**Figure 6:**
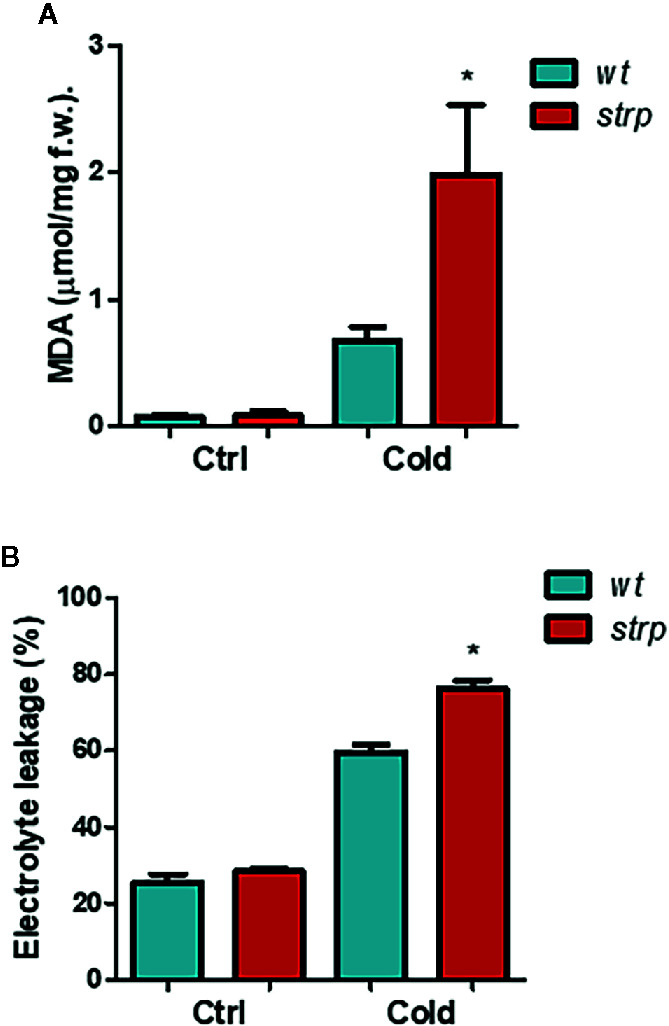
The s*trp* mutation increases the cold-induced oxidative damages in *A. thaliana.* *Wild type* and *strp* mutant plants were exposed to 4°C for 18 h. Leaves were then used to assay MDA production (Panel **A**) and ion leakage (Panel **B**). Error bars are S.E.M. of three independent experiments. *P < 0.01, by Student’s t-test.

As shown in **Figure 6A**, MDA content is strongly increased by cold stress both in *wild type* and in *strp* plants. However, MDA production is significantly higher in the mutant as compared to *wild type*.

Besides lipid peroxidation, cold stress also affects membrane integrity, as a result of ROS-induced or mechanical damages. Relative electrolyte conductivity (REC), corresponding to membrane permeability to ions, was measured in leaves of cold-treated *wild type* and *strp* plants. As shown in **Figure 6B**, cold stress induces a significant increase in the ion leakage, both in *wild type* and *strp* mutant. Membrane damages are significantly higher in the mutant as compared to *wild type*. These results demonstrate that cold-induced oxidative damages have led to a more severe loss of membrane functionality in the *strp* mutant.

### STRP Levels in Response to Exogenous ABA

**Figure 7:**
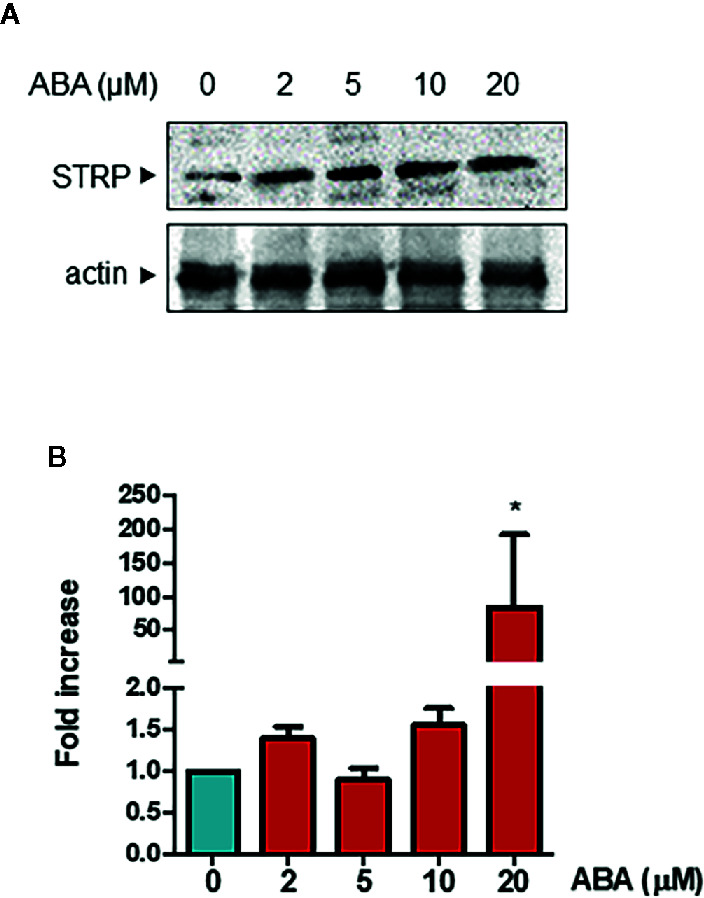
ABA treatment activates *STRP* expression and increases STRP levels. STRP levels and gene expression were assessed on 2-week-old seedlings treated with 2, 5, 10, and 20 µM ABA for 18 h. STRP levels were determined by western blot (Panel **A**) on 20 μg of total extract. Samples were separated by SDS-PAGE, transferred on PVDF membrane and incubated with the anti-STRP antibodies. Actin was used as loading control. *STRP* expression levels (Panel **B**) were determined on total RNA extracted from *A. thaliana*, retrotranscribed and analysed by RT-qPCR. mRNA levels were normalized to beta 8-tubulin mRNA (*TUB8*). Error bars are S.E.M. of four independent experiments. *P < 0.01, by Student’s t-test.

It is well known that ABA plays a significant role in plant responses to cold stress (Cutler et al., 2010) and that ABA levels are increased under low temperature conditions (Lång et al., 1994). Hence, the ability of the hormone to influence STRP protein levels was investigated. Two-week-old plants were treated with different ABA concentrations for 18 h, and STRP levels were analysed by western blot. As shown in **Figure 7A**, all ABA concentrations tested induce a strong increase of STRP levels. To ascertain whether STRP increase depends on gene activation, total RNA was extracted from ABA-treated plants, and *STRP* expression was evaluated by RT-qPCR. As shown in **Figure 7B**, *STRP* expression is only induced at the highest ABA concentration, whereas increase of protein levels occurs also at lower concentrations, suggesting the presence of different mechanisms regulating STRP levels in response to ABA.

### Effects of ABA on *strp* Growth and Development

**Figure 8:**
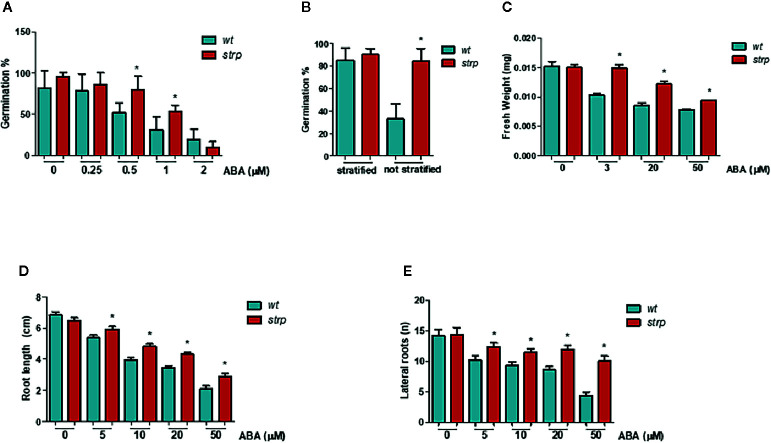
The *strp* mutant has an ABA-hyposensitive phenotype. **(A)** germination rate of *strp* and *wild type* seeds, assessed on seeds sowed on MS supplemented with 0.25, 0.5, 1, and 2 µM ABA, stratified and transferred for one week in the growth chamber. **(B)** germination after stratification or not, determined on 200 *wild type* and *strp* seeds after one week from sowing on MS. **(C)** plant growth rate on ABA, analysed on *strp* and *wild type* seedlings sowed on MS and transferred after 4 days on MS supplemented with 3, 20, and 50 µM ABA, and grown for additional two weeks. About 50 seedlings for each ABA concentration were used. **(D, E)** primary root elongation and lateral roots formation, assessed on *wild type* and *strp* plants germinated on MS and transferred after 4 days on the same medium supplemented with 5, 10, 20, and 50 µM ABA and grown for one additional week. Error bars are S.E.M. of three independent experiments. *P < 0.01, by Student’s t-test.

Since ABA treatment strongly increased STRP levels, the effect of *STRP* mutation on different ABA-regulated processes was investigated. The inhibitory effect of ABA on seed germination was evaluated by treating *strp* and *wild type* seeds with different ABA concentrations. As shown in **Figure 8A**, the germination rate of *strp* seeds is significantly higher in the presence of 0.5 µM and 1 µM ABA, thus suggesting that the mutant is hyposensitive to ABA. At the highest ABA concentration tested (2 µM), the mutant is no longer able to counteract the inhibitory action of the hormone and responds similarly to the *wild type*.

To determine whether the *strp* mutant is also hyposensitive to endogenous ABA, the germination efficiency in the absence of exogenous ABA was analysed. As shown in **Figure 8B**, the germination rate of *wild type* seeds is strongly reduced in the absence of stratification. Interestingly, the *strp* mutant maintains instead a high germination rate. This result suggests that the mutant is also hyposensitive to the inhibition of germination exerted by endogenous ABA.

To test the effect of *strp* mutation in the ABA-mediated inhibition of plant growth, *wild type* and *strp* plants were grown in MS agar plates in the presence of different concentrations of ABA. The weight of the seedling was measured after two weeks, while the length of primary root and the number of lateral roots were measured after one week of ABA treatment. For this experiment, *wild type* and *strp* plants of the same weight were utilized. Hormone addition in the growth medium strongly reduces the weight of both *wild type* and *strp* plants, with a less pronounced effect in the mutant (**Figure 8C**). Moreover, roots of the *strp* mutant are less sensitive than *wild type* to the inhibitory effect of exogenous ABA, both in terms of primary root growth (**Figure 8D**) and of lateral root development (**Figure 8E**). Together, these results indicate that the *strp* mutant is hyposensitive to ABA effects on plant development.

**Figure 9:**
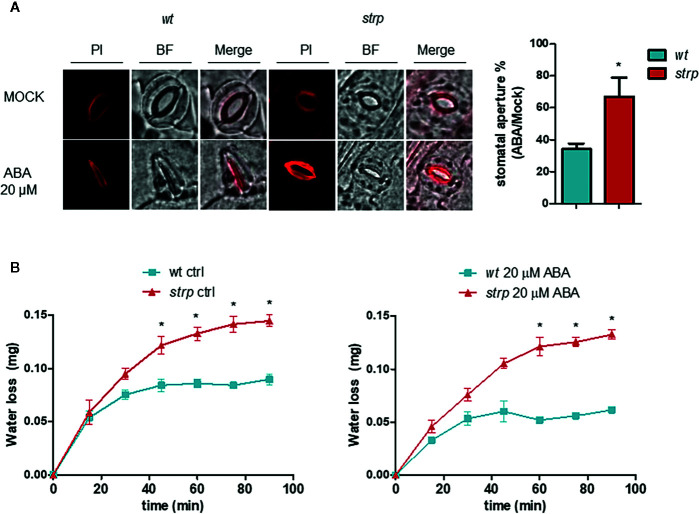
The s*trp* mutation alters the ABA-induced stomatal closure and increases water loss by transpiration. **(A)** ABA-mediated stomatal closure, evaluated by treating the leaves with 20 µM ABA for 2 h. Epidermis was then isolated and stained with propidium iodide. **(B)** water loss was detected on *strp* and *wild type* detached leaves treated with 20 µM of ABA. Data are represented as variation of fresh weight respect to T0. Error bars are S.E.M. of three independent experiments. *P < 0.01, by Student’s t-test.

The ability of ABA to induce stomatal closure was also investigated, using detached leaves from three-week-old *wild type* and *strp* plants. Leaves were first incubated with an appropriate solution for stomata opening, then treated for 2 h with 20 µM ABA. Epidermal peels were then isolated and stained with Propidium Iodide (PI). As shown in **Figure 9A**, ABA-induced stomatal closure is significantly reduced in the *strp* mutant.

A reduced stomatal closure in response to ABA should result in increased water loss through transpiration. Hence, water loss was determined using detached *wild type* and *strp* leaves treated with 20 µM ABA. As shown in **Figure 9B**, *strp* mutant leaves exhibit an increased leaf water loss relative to the *wild type*, both in the absence or in the presence of ABA.

Taken together, these results demonstrate that the *strp* mutant is hyposensitive to all the effects mediated by ABA on plant growth, development and in the responses to drought stress, thus proposing the involvement of STRP in ABA signalling or homeostasis.

### Expression Levels of Genes Involved in ABA Signalling and Biosynthesis in the *strp* Mutant

**Figure 10:**
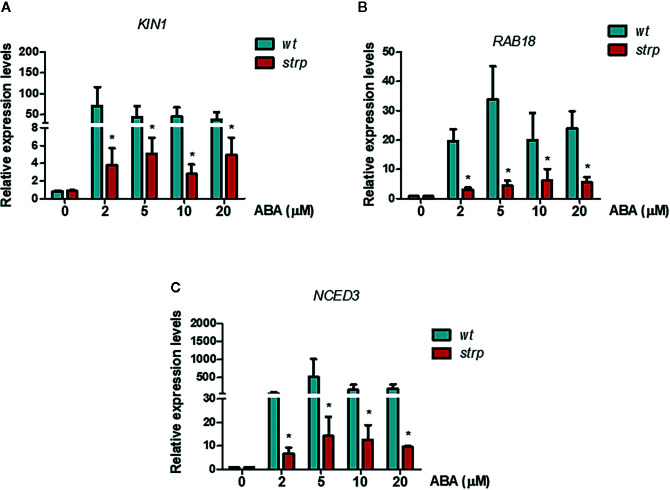
ABA-responsive genes expression is reduced in the *strp* mutant. Relative expression of *KIN1* **(A)**, *RAB18* **(B)**, and *NCED3* **(C)** was determined on two-week-old *wild type* and *strp* plants treated with 2, 5, 10, and 20 µM ABA for 18 h. mRNA levels were normalized to actin mRNA (*ACT8*). Error bars are S.E.M. of three independent experiments. *P < 0.01, by Student’s t-test.

To investigate the possible role of STRP in ABA signalling, the transcriptional activation of the ABA-responsive genes *KIN1* and *RAB18* was assessed by RT-qPCR. As expected, exogenous ABA activates *KIN1* and *RAB18* expression in *wild type* plants (**Figures 10A, B**). As shown in **Figures 10A, B**, the activation of these genes in the *strp* mutant is strongly reduced at all the ABA concentrations tested. The impaired activation of the ABA-responsive genes in the mutant suggests that the reduced ABA responsiveness could be ascribable to a downregulation of ABA signalling pathway.

A key role in ABA biosynthesis is played by the 9-cis-epoxycarotenoid dioxygenase (NCED). Oxidative cleavage of epoxy-carotenoids is a highly regulated step, and ABA accumulation is the result of the stimulation of *NCED* expression in response to various stresses (Xiong and Zhu, 2003). Moreover, a positive feedback mechanism for ABA-mediated activation of its biosynthetic genes, including *NCED3*, is also present. Hence, activation of *NCED3* gene in response to exogenous ABA was analysed in the *strp* mutant by RT-qPCR. As shown in **Figure 10C**, *NCED3* is strongly activated by ABA in *wild type* plants, whilst the expression of the gene is greatly impaired in the *strp* mutant, suggesting that also the endogenous levels of ABA can be impaired in the *strp* mutant.

## Discussion

The aim of the present work was to understand the role played by STRP protein in the response mechanisms to cold stress in *A. thaliana*. The evidence that STRP levels strongly increased in total cellular extracts of plants exposed to low temperature (Rocco et al., 2013) prompted us to perform an extensive biochemical characterization of the protein. Western blot analysis showed that STRP rapidly increases under cold stress into the cytosol. Confocal microscopy and sub-cellular fractionation experiments suggest that the protein could be also associated to the plasma membrane, and that cold stress brings about a reduction of protein content localized in this compartment. In this scenario, STRP could act as a stress sensor, since plasma membrane-associated proteins can perceive extracellular stimuli (e.g. low temperature), transducing them in an intracellular response. At the plasma membrane level, STRP could be associated to the phospholipid bilayer or, alternatively, to a membrane docking protein. Similar mechanisms are rather recurring in plants: several stress sensors are anchored to plasma membrane proteins or lipids, e.g. to sphingolipids, sterols, and glycosylphosphatidylinositol (Niu and Xiang, 2018). On the other hand, different proteins, such as Heat Shock Proteins, G proteins, and phospholipase C and D, are well-known membrane-linked stress sensors (Plieth et al., 1999; Ruelland et al., 2002; Gupta et al., 2011; Guo et al., 2011). Hence, the release of STRP from the membrane could be part of a transduction pathway leading to cold stress tolerance.

As far as the mechanism underlying the increase of STRP levels, it was found that *STRP* gene is not induced under cold stress conditions, suggesting a different regulatory mechanism. In non-stressed plants, STRP protein levels increased by a proteasome inhibitor treatment, indicating that STRP is targeted for degradation by the ubiquitin-proteasome system and that cold stress stabilizes the protein by inhibiting this process.

STRP also localizes in the nucleus, as ascertained by western blot and STRP-YFP transient expression in *A. thaliana* protoplasts. Cold stress determines the increase of STRP nuclear content, promoting protein association to chromatin. It is well known that chromatin organization plays a key role in stress responses (Kim et al., 2010; Zhu et al., 2012; Han and Wagner, 2014). The recent finding that the chromatin-remodelling protein DEK3 interacts with STRP (Waidmann et al., 2014) strongly proposes the involvement of STRP in chromatin remodelling processes under cold stress, modulating the expression of specific cold-activated genes. In addition, cold stress induces oxidative damages to the main cellular structures, including DNA (Apel and Hirt, 2004; Bartoli et al., 2004). Hence, STRP interaction with chromatin could also represent a protection mechanism in response to ROS.

STRP has no similarity with other well-characterized *A. thaliana* proteins. However, STRP has high similarity with WCI16, a wheat LEA-like protein (Sasaki et al., 2014). Correspondingly, STRP is a high hydrophilic unstructured protein, with just few regions predicted to be folded in a context-dependent manner. Moreover, STRP is highly soluble after boiling and is a good cryoprotectant, able to prevent enzymes inactivation after freezing. Hence, despite the absence of canonical LEA motifs in the STRP sequence, the protein shares some common features with LEA proteins. Consequently, STRP accumulation under cold stress could greatly contribute to stabilize cellular membranes, to prevent protein misfolding and therefore to preserve enzyme activities.

Phenotypical analysis of the *strp* knockout insertional mutant revealed that the mutant has an impaired growth, with short hypocotyls and reduced leaf area. Cold stress hampered the growth rate of rosette leaves similarly in *wt* and in *strp* plants. However, the *strp* mutant shows an increased stress-induced oxidative damage, suggesting the participation of STRP in ROS-detoxifying mechanisms during cold stress acclimation. Generally, oxidative stress and membrane damage lead to plant growth inhibition (Gechev et al., 2006). It is therefore conceivable that, in the stress conditions used, the production of ROS and the extent of lipid peroxidation is too low to induce a significant growth defect.

Considering the role played by ABA in a range of abiotic stresses, a possible connection between ABA and the STRP-mediated response to cold stress was analysed. Exogenous ABA application on *A. thaliana* brings about a strong accumulation of STRP protein, and high ABA concentrations induce *STRP* transcriptional activation. The analysis of ABA effects on *strp* revealed that the mutant is hyposensitive to all the effects of ABA on plant development. In fact, the mutant has a longer primary root, increased lateral roots formation, high germination rate (0.5 and 1 µM), even without stratification, altered stomatal closure and increased water loss under water deficit. Moreover, down-regulation of the ABA-responsive genes further confirms that ABA responses are impaired in the *strp* mutant.

The interplay between STRP function and ABA remains to be clarified, being not clear how STRP is correlated to the hormone signalling and perception. Hence, the characterization of STRP-mediated stress responses in ABA-deficient mutant could contribute to clarify this aspect.

In conclusion, results obtained identify STRP as a multitasking protein acting at different levels in the response mechanisms to low temperatures in *A. thaliana*. Moreover, this work lays the foundation for the clarification of a possible involvement of the protein in the resistance mechanism toward different abiotic and biotic stress.

## Data Availability Statement

The raw data supporting the conclusions of this article will be made available by the authors, without undue reservation.

## Ethics Statement

Ethical review and approval was not required for the animal study because The rabbit antibody production has been carried out following the guidelines of the Institutional Animal Care and Use Committee (IACUC) of the Interdepartmental Service Center - Station for Animal Technology of the University of Rome Tor Vergata, in compliance with the Legislative Decree of 27 January 1992 n. 116, which implements the EEC directive n. 609/86.

## Author Contributions

LC, SV, and AF formulated the original hypothesis of the article and designed the experiments. AF and SV performed the experiments. MM prepared the anti-STRP antibody. LC and AF wrote the manuscript and prepared the figures. PA analysed the data and revised the manuscript. All authors contributed to the article and approved the submitted version.

## Conflict of Interest

The authors declare that the research was conducted in the absence of any commercial or financial relationships that could be construed as a potential conflict of interest.

## Supplementary Material

Figure S1 SDS-PAGE and Western blot analysis on STRP. To test the ability of the anti-STRP polyclonal antibody to recognize STRP, 1 µg of purified recombinant protein and 15 μg of plant whole cell extract (W.C.E.) were separated by 12% SDS-PAGE, transferred on PVDF membrane and incubated with the anti-STRP antibodies. CBB, Coomassie Brilliant Blue; WB, western blot.

Figure S2 The *strp* mutant is homozygous for the T-DNA insertion, leading to loss of *STRP* expression. A: left panel, schematic model of the T-DNA insertion in the STRP promoter; right, genomic three-oligonucleotides PCR for the identification of homozygous mutant lines. Genotype analysis of the *strp* mutant was carried out on two different plant generations, named T2 and T3, with a genomic PCR using three primers: LP primer 5’-TACACTCACTCGTCACTCCCC-3’, RP primer 5’-TAAATGTCTTTTTCGGCAACG-3’ and LBb1.3 primer 5’-ATTTTGCCGATTTCGGAAC-3’. The genomic DNA extraction was performed with the “DNeasy^®^ Plant Mini Kit” (QIAGEN) according to manufacturer’s instructions. The reaction with LB+RP, complementary to the STRP promoter, and with BP+RP, complementary to the T-DNA insertion and the STRP promoter, were programmed to get the blank for homozygous mutant and for *wild type* plants, respectively. The complete reaction with the three oligonucleotides (LP+BP+RP) produces one amplified fragment of 900 bp for the *wild type* and one of 700 bp for the homozygous mutant. B: RT-PCR (left panel) and RT-qPCR (right panel) were used to verify the lack of *STRP* expression in the *strp* mutant. The PCR were performed with the following primer pairs: 5’-CCATCTCTTAACTCTTCCATCCAA-3’, and 5’-TCTGGCTCCTCTGGTGTT-3’. mRNA levels were normalized to GAPDH mRNA. Error bars are S.E.M. of three independent experiments. *P < 0.01, by Student’s t-test. C: western blot analysis, performed on 20 µg of W.C.E. of *wild type* and *strp* T2 and T3 plants. Samples were separated by 12% SDS-PAGE, transferred on PVDF and incubated with anti-STRP antibodies.

Figure S3 Densitometric analysis of bands of western blot experiments. Densitometric analysis was performed using the ImageJ image-processing program. Data are expressed as the percentage of the maximum integrated densitometric value (IDV, the product of the area and mean grey value).
