# Supplementary figures and images for: The Salt Tolerance Related Protein (STRP) Mediates Cold Stress Responses and Abscisic Acid Signalling in Arabidopsis thaliana
Source: Front Plant Sci. 2020 Aug 13;11:1251. doi: 10.3389/fpls.2020.01251 (PMC7438554; doi:10.3389/fpls.2020.01251)

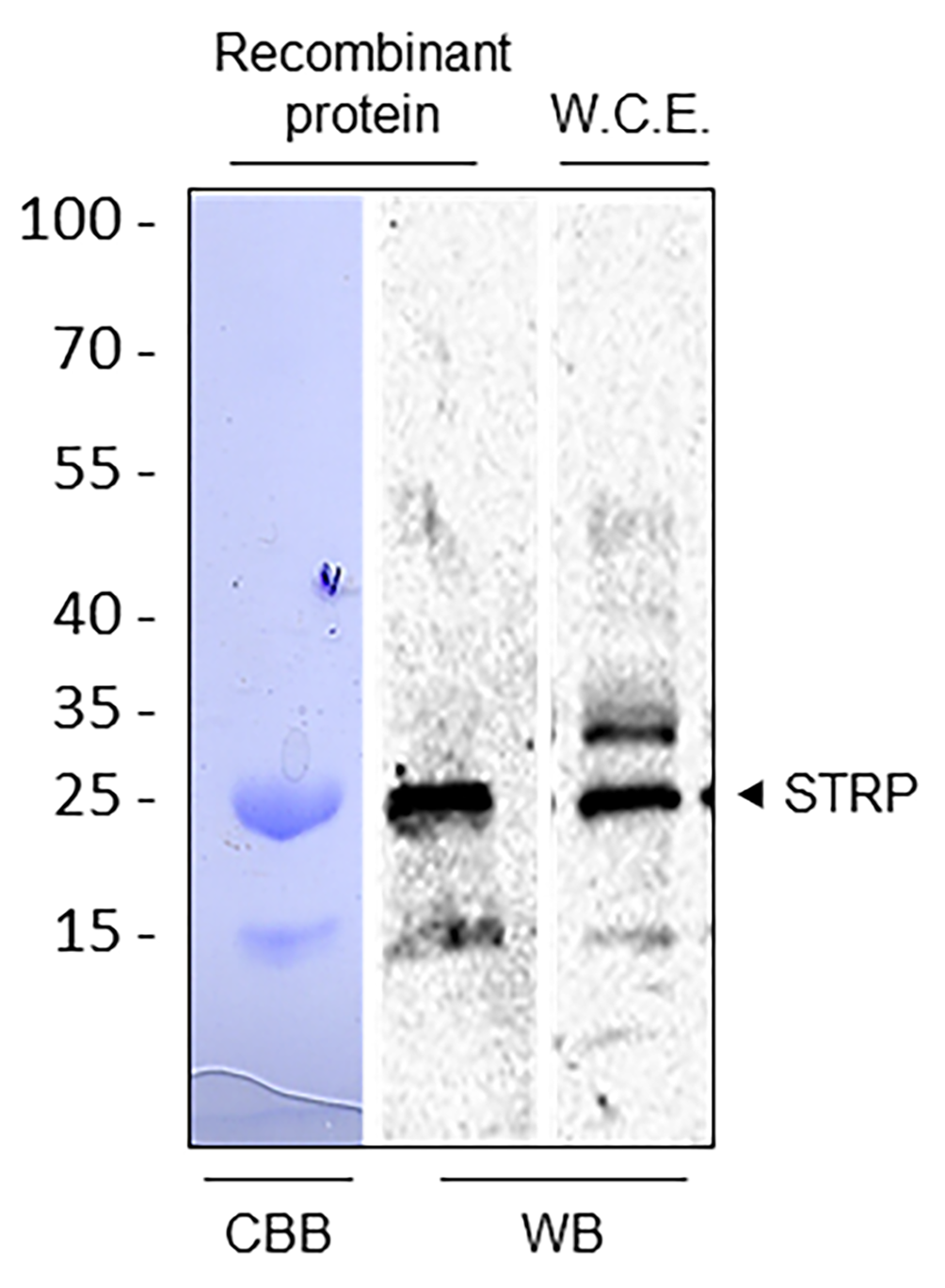

Supplement: Figure S1 — SDS-PAGE and Western blot analysis on STRP. To test the ability of the anti-STRP polyclonal antibody to recognize STRP, 1 µg of purified recombinant protein and 15 μg of plant whole cell extract (W.C.E.) were separated by 12% SDS-PAGE, transferred on PVDF membrane and incubated with the anti-STRP antibodies. CBB, Coomassie Brilliant Blue; WB, western blot. [file Image_1.tif]

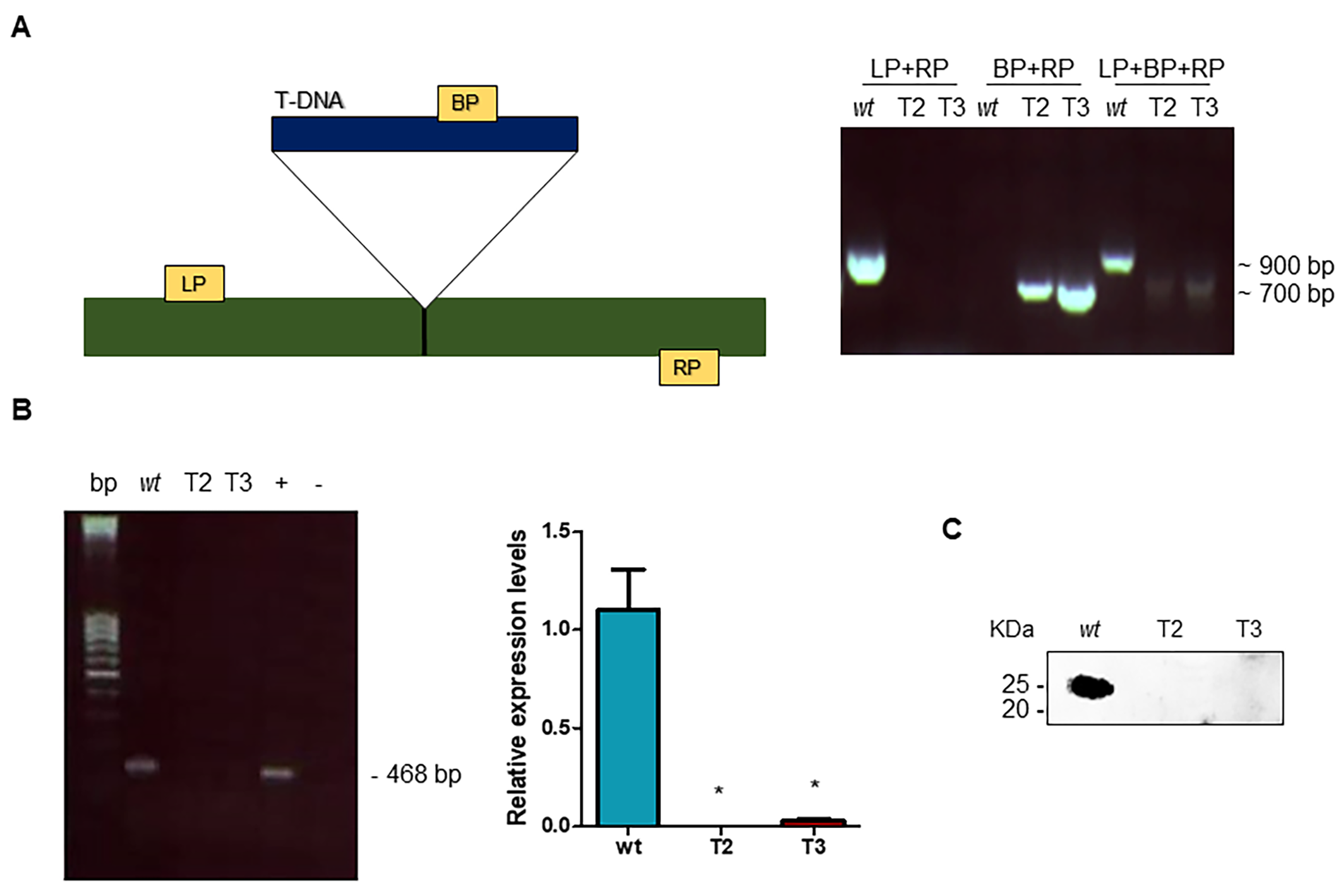

Supplement: Figure S2 — The strp mutant is homozygous for the T-DNA insertion, leading to loss of STRP expression. A: left panel, schematic model of the T-DNA insertion in the STRP promoter; right, genomic three-oligonucleotides PCR for the identification of homozygous mutant lines. Genotype analysis of the strp mutant was carried out on two different plant generations, named T2 and T3, with a genomic PCR using three primers: LP primer 5’-TACACTCACTCGTCACTCCCC-3’, RP primer 5’-TAAATGTCTTTTTCGGCAACG-3’ and LBb1.3 primer 5’-ATTTTGCCGATTTCGGAAC-3’. The genomic DNA extraction was performed with the “DNeasy® Plant Mini Kit” (QIAGEN) according to manufacturer’s instructions. The reaction with LB+RP, complementary to the STRP promoter, and with BP+RP, complementary to the T-DNA insertion and the STRP promoter, were programmed to get the blank for homozygous mutant and for wild type plants, respectively. The complete reaction with the three oligonucleotides (LP+BP+RP) produces one amplified fragment of 900 bp for the wild type and one of 700 bp for the homozygous mutant. B: RT-PCR (left panel) and RT-qPCR (right panel) were used to verify the lack of STRP expression in the strp mutant. The PCR were performed with the following primer pairs: 5’-CCATCTCTTAACTCTTCCATCCAA-3’, and 5’-TCTGGCTCCTCTGGTGTT-3’. mRNA levels were normalized to GAPDH mRNA. Error bars are S.E.M. of three independent experiments. *P < 0.01, by Student’s t-test. C: western blot analysis, performed on 20 µg of W.C.E. of wild type and strp T2 and T3 plants. Samples were separated by 12% SDS-PAGE, transferred on PVDF and incubated with anti-STRP antibodies. [file Image_2.tif]

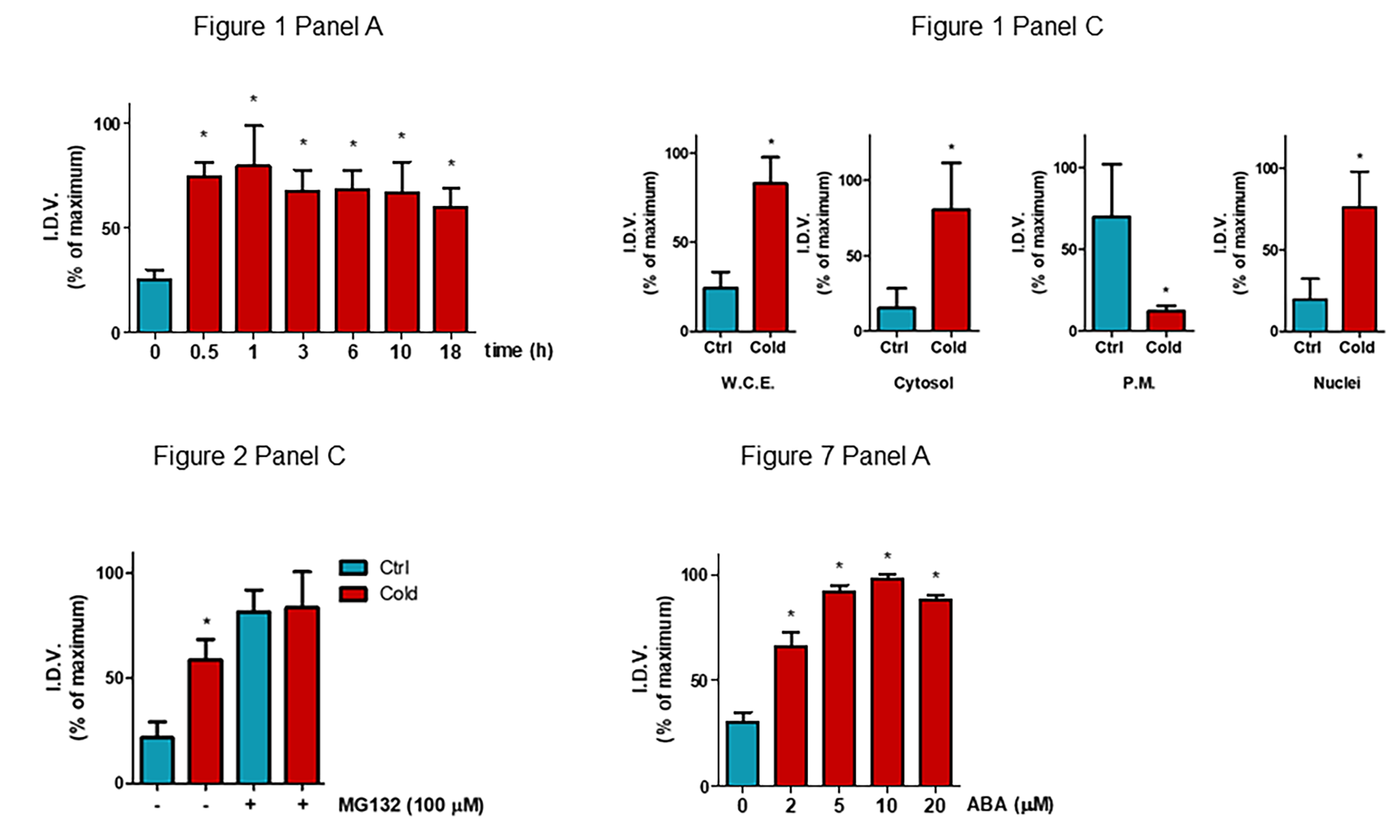

Supplement: Figure S3 — Densitometric analysis of bands of western blot experiments. Densitometric analysis was performed using the ImageJ image-processing program. Data are expressed as the percentage of the maximum integrated densitometric value (IDV, the product of the area and mean grey value). [file Image_3.tif]
